# Educating Substance Use Treatment Center Providers on Tobacco Use Treatments Is Associated with Increased Provision of Counseling and Medication to Patients Who Use Tobacco

**DOI:** 10.3390/ijerph20054013

**Published:** 2023-02-23

**Authors:** Brian J. Carter, Ammar D. Siddiqi, Tzuan A. Chen, Maggie Britton, Isabel Martinez Leal, Virmarie Correa-Fernández, Anastasia Rogova, Bryce Kyburz, Teresa Williams, Kathleen Casey, Lorraine R. Reitzel

**Affiliations:** 1 Department of Health Disparities Research, The University of Texas MD Anderson Cancer Center, 1400 Pressler St., Houston, TX 77030, USA; 2 Department of Psychological, Health, and Learning Sciences, University of Houston, 3657 Cullen Blvd, Stephen Power Farish Hall, Houston, TX 77204, USA; 3 Department of Biosciences, Rice University, 6100 Main St., Houston, TX 77005, USA; 4 HEALTH Research Institute, University of Houston, 4349 Martin Luther King Blvd, Houston, TX 77204, USA; 5 Integral Care, 1430 Collier St., Austin, TX 78704, USA

**Keywords:** counseling, implementation science, medication, perceived barriers, pharmacotherapy, substance use treatment centers, tobacco cessation, tobacco use treatment, tobacco-free workplace programs, knowledge barrier

## Abstract

Tobacco use is the leading preventable cause of death in America and is elevated among patients with non-tobacco substance use disorders. Substance use treatment centers (SUTCs) do not commonly address their patients’ tobacco use. Lack of knowledge on treating tobacco use with counseling and medication may be a barrier that underlies this inaction. A multi-component tobacco-free workplace program implemented in Texas SUTCs educated providers on treating tobacco use with evidence-based medication (or referral) and counseling. This study examined how center-level changes in knowledge from pre- to post-implementation (i.e., over time) affected center-level behavioral changes in providers’ provision of tobacco use treatment over time. Providers from 15 SUTCs completed pre- and post-implementation surveys (pre N = 259; post N = 194) assessing (1) perceived barriers to treating tobacco use, specifically, a lack of knowledge on treating tobacco use with counseling or medication; (2) receipt of past-year education on treating tobacco use with counseling or medication; and (3) their intervention practices, specifically, the self-reported regular use of (a) counseling or (b) medication intervention or referral with patients who use tobacco. Generalized linear mixed models explored associations between provider-reported knowledge barriers, education receipt, and intervention practices over time. Overall, recent counseling education receipt was endorsed by 32.00% versus 70.21% of providers from pre- to post-implementation; the regular use of counseling to treat tobacco use was endorsed by 19.31% versus 28.87% from pre- to post-implementation. Recent medication education receipt was endorsed by 20.46% versus 71.88% of providers from pre- to post-implementation; the regular use of medication to treat tobacco use was endorsed by 31.66% versus 55.15% from pre- to post-implementation. All changes were statistically significant (*p*s < 0.05). High versus low reductions in the provider-reported barrier of “lack of knowledge on pharmacotherapy treatment” over time were a significant moderator of effects, such that SUTCs with high reductions in this barrier were more likely to report greater increases in both medication education receipt and medication treatment/referral for patients who use tobacco over time. In conclusion, a tobacco-free workplace program implementation strategy that included SUTC provider education improved knowledge and resulted in increased delivery of evidence-based treatment of tobacco use at SUTCs; however, treatment provision rates—in particular, offering tobacco cessation counseling—remained less than desirable, suggesting that barriers beyond lack of knowledge may be important to address to improve tobacco use care in SUTCs. Moderation results suggest (1) differences in the mechanisms underlying uptake of counseling education versus medication education and (2) that the relative difficulty of providing counseling versus providing medication persists regardless of knowledge gains.

## 1. Introduction

Tobacco use is the leading cause of preventable death, disease, and disability in the United States (U.S.) [1]. It has been causally linked to myriad chronic diseases, including cancer, stroke, diabetes, chronic obstructive pulmonary disease, and respiratory diseases [2]. Chronic nicotine exposure has a deleterious impact on memory and cognition [3]. Despite the known health effects, nearly 31 million U.S. adults smoke cigarettes, the most common form of tobacco use, and millions of others use non-cigarette tobacco products (e.g., e-cigarettes and smokeless tobacco) [1]. Most adults who smoke recognize the detrimental health effects of smoking and want to quit [4,5]. However, of the 68% who report wanting to quit smoking each year [4], only 7.4% succeed [5]. This is in part because (a) nicotine exposure alters the brain’s signaling pathways to promote continued tobacco product use [3] and (b) the tobacco industry has a propensity to employ pernicious marketing campaigns designed to make its products both appealing and easily accessible [6]. The continued widespread use of tobacco costs the U.S. USD 600 billion annually in increased healthcare spending and lost productivity [7].

Tobacco control efforts have been successful in reducing tobacco use overall in the U.S.; however, some population subgroups, such as adults with non-nicotine substance use disorders (SUDs), continue to use tobacco at high rates [8,9,10]. For example, compared to those without SUDs, adults who reported a SUD within the past year were 2 times more likely to smoke cigarettes [11]. Moreover, 65–87% of adults receiving addiction treatment report smoking [12]. As a result, adults with SUDs are disproportionately burdened with tobacco-related disease and premature death relative to the general population [11,13]. A 2015 study examined death records of 148,761 deceased Oregon residents and found that adults with SUDs had higher rates of tobacco-related deaths than did the population as a whole (53.6% vs. 30.7%) [13]. Furthermore, more adults with SUDs died from tobacco-related causes than from all other causes combined [13]. Consequently, targeted intervention efforts are needed to extend the reach of evidence-based tobacco use care to mitigate tobacco-related health inequities among adults with SUDs.

The U.S. Preventive Services Taskforce and other clinical guidelines recommend that patients be screened for tobacco use at every healthcare encounter [14] and provided with evidence-based cessation care [15,16]. Recommended behavioral interventions include brief advice and individual or group counseling [14,16], and recommended pharmacotherapy includes nicotine-based medications (nicotine replacement therapy (NRT)) or non-nicotine medications, such as bupropion and varenicline (NRT and non-nicotine medications, collectively, “medication”) [14,16]. However, provider engagement in these recommended practices is not ubiquitous at substance use treatment centers (SUTCs), where patients with SUDs receive care. One nationwide study showed that only 64% of SUTCs performed tobacco screenings, 47% provided tobacco cessation counseling to patients, and between 20% and 26% offered medication, dependent on type (NRT vs. non-nicotine medication) [17]. There is evidence that tobacco use is prevalent among SUTC staff (20.4–32.0% currently use tobacco and 47.9–48.8% formerly used tobacco) and that SUTC staff who use tobacco are significantly less likely to intervene in patients’ tobacco use [18,19]. These low tobacco care provision rates represent a missed opportunity to address the elevated tobacco use and consequent health disparities among adults with SUDs who receive care in SUTCs.

Various theories (e.g., Theory of Planned Behavior, Knowledge–Attitude–Practice Theory) and organizational/implementation frameworks (e.g., Theoretical Domains Framework) suggest that knowledge may be a key determinant of healthcare providers’ behaviors [20,21,22]. Indeed, numerous studies support that misconceptions (i.e., a lack of accurate knowledge) may underlie SUTC providers’ underuse of evidence-based intervention to address patients’ tobacco use [17,23,24,25,26]. For example, despite empirical evidence to the contrary [27,28,29,30,31], SUTC providers report beliefs that tobacco cessation interferes with alcohol or other drug sobriety [17,23], that tobacco cessation medication may be harmful to patients [24], that tobacco use is a helpful coping strategy and a facilitator of patients’ social connections [26], and that addressing tobacco use is less important than addressing other SUDs [17,25]. Other commonly reported SUTC provider knowledge gaps include how to treat tobacco use with counseling [24,25] and how to comfortably talk to patients about quitting [24]. Therefore, interventions addressing SUTC providers’ knowledge gaps through provider education may reduce their perceived barriers to providing tobacco use care and enhance the implementation of evidence-based recommendations in these settings [20,32,33,34,35].

Studies support that educational interventions can change providers’ attitudes about treating tobacco use, improve knowledge of tobacco use care, and enhance the provision of tobacco use care to patients in various healthcare settings [36,37,38]. However, it is unclear whether education is sufficient to enhance all types of tobacco care provision (i.e., counseling versus medication use/recommendation) in SUTCs. Gaps in the literature exist on this point. For example, prior studies on training healthcare providers to treat tobacco use fail to specifically educate them about medications, and/or measure changes in providers’ use of medications from before to after education [36,37,39]. Similarly, work conducted with substance use treatment providers either does not assess if education addressed knowledge barriers that interfered with providers’ tobacco use care provision to patients and/or the links between education provision and subsequent changes in counseling and medication practices [40,41]. Understanding more about these links, however, may be helpful for tailoring educational initiatives for maximal actualization in providers’ work with patients.

The actualization of SUTC provider education into changes in providers’ use of tobacco counseling versus tobacco medication use/recommendation with SUD patients may differ for several reasons. For example, most SUTC providers without medical degrees have advanced training in non-nicotine addiction counseling—skills that should be transferrable to tobacco use treatment but do not seem to be routinely employed in that regard. Consequently, addressing misperceptions about tobacco use co-treatment and making explicit the transferability of extant counseling skills *should* directly translate into enhanced tobacco use counseling with patients. However, it is possible that delivery of tobacco use counseling may be relatively unaltered following education due to perceived limitations in time [24,25], skills [42,43,44], and/or comfort with care provision [24,45] (i.e., barriers to treatment distinct from knowledge). Relative to counseling provision, NRT provision or non-nicotine medicine recommendation may be more resilient to these barriers inasmuch as it is more straightforward (provider information provision versus patient–provider dialogue) and thus less time-consuming, resulting in greater uptake by providers. Moreover, exposure to education about how tobacco use can be treated with medications may be more novel to the vast majority of providers in SUTCs, who are not medically trained and would typically have less knowledge, training, and experience with recommending medications to patients [46,47]. Tobacco cessation care information novelty has been linked to the greater valuation of its provision by treatment center leadership [48], greater provider knowledge gain from education [49], and larger center-level increases in tobacco use care provision to patients, though the relative effects on counseling versus medication care provision were not studied. However, medication care provision is also less prevalent than cessation counseling in SUTCs [17] and thus has greater potential to significantly increase post-education receipt (due to ceiling effects). It may also be the case that medication care/referral provision may not change with increased knowledge if other barriers continue to exist. Overall, more research is needed to understand the effects of educational interventions on SUTC providers’ endorsement of knowledge barriers to tobacco use care provision and, ultimately, their provision of cessation counseling and medication to patients [41], as increased provision of each is aligned with best practices [15,16]. Moreover, examining the effects of education receipt on provider care at the SUTC level is of interest given that SUTC practices can affect the tobacco use prevalence in an entire catchment area, particularly in more rural areas where access to care is sparse and tobacco-related health disparities may be more common [50]. This information can help to tailor time-limited educational interventions to an SUTC’s needs (e.g., relative focus on cessation counseling information vs. medication information, barriers to counseling other than knowledge limitations), maximizing its potential to impact tobacco use disparities in a given community.

The present study addresses the aforementioned gaps in the literature by examining how education provision affected changes in providers’ self-reported treatment knowledge barriers and their provision of tobacco cessation counseling and medication within 15 SUTCs in Texas that participated in a comprehensive tobacco-free workplace program. Provider education, theoretically linked to knowledge barriers and treatment provision, was a core component of the program. Despite the dearth of prior studies to inform definitive hypotheses about all associations, based on theories [20,21,22] and our empirical and anecdotal program implementation observations, we expected that (a) prior to program implementation, knowledge gaps hindering medication intervention/referral provision would be more substantial than knowledge gaps hindering cessation counseling provision; (b) program implementation would reduce providers’ self-reported knowledge barriers for both cessation medication and counseling care provision and (c) enhance the delivery of patient care in each area at the SUTC level, with (d) greater changes seen for provision of medication than counseling provision; and (e) the magnitude of changes at the SUTC level in knowledge barriers (low versus high) over time consequent to education receipt would moderate changes in the use of counseling and medication provision/referral for SUD patients who used tobacco. This analysis was retrospective; however, findings may inform future efforts to implement tobacco-free workplace programs with provider education at SUTCs and to identify which SUTCs are most ripe for such implementation, in each case, to maximize delivery of evidence-based tobacco use intervention to patients.

## 2. Materials and Methods

### 2.1. Participants and Recruitment

Participants were direct service providers (e.g., physicians, medical assistants, nurses, nursing assistants, qualified mental health professionals, or licensed chemical dependency counselors; each, hereafter, a “provider”) at 15 SUTCs that completed Taking Texas Tobacco Free (TTTF), a comprehensive tobacco-free workplace program, from December 2017 to May 2020. Recruitment for TTTF was specific to Texas due to funder requirements and was primarily accomplished via direct email solicitation and word of mouth. Decisions about participation were made by each center’s leadership/CEO. TTTF enrollment was ongoing such that each center received the same intervention components over a period of 7.2 to 13.6 (10.96 + 3.84) months, negotiated based on each center’s capacity for prompt implementation. Together, the SUTCs that participated in this study provided services for the following counties: Harris, Tarrant, Victoria, Bexar, Nueces, Travis, Grayson, Dallas, and Galveston. Collectively, they reported serving over 80,000 patients across approximately 300,000 annual visits. The TTTF program is further detailed in prior publications [48,49,51,52,53,54,55,56,57,58,59,60,61,62]; characteristics of participating SUTCs were also previously reported [63].

### 2.2. Program Implementation

The TTTF program entailed the delivery of a ~1.5 h education session to providers that included a review of the health risks of tobacco use, disparities experienced by patients with substance use dependencies, the use of brief interventions for screening and treating tobacco use, the importance and beneficial outcomes of co-treating tobacco use within substance use dependency treatment, and how to use NRT and non-nicotine medications to treat tobacco use disorder. “Program champions”, providers identified by center leadership to work with TTTF staff in program implementation, attended a 5-day educational course accredited to certify them as tobacco treatment specialists with the expertise to later serve as a resource to other providers at their SUTCs. Program champions did not receive additional financial compensation for their services. Centers generally had between 1–3 program champions, dependent upon center size and as negotiated with center leadership. Program champions and center providers were also invited to attend a ~7.5 h motivational interviewing educational workshop delivered by TTTF staff, with varying uptake by center. Each SUTC participating in TTTF received a starter kit of NRTs for patients and employees and was encouraged to continue to budget for these products themselves so that they could be offered free of charge to patients. Each participating center also implemented a comprehensive tobacco-free workplace policy that disallowed tobacco product use indoors or anywhere on the premises. The TTTF program provided each center with permanent tobacco-free workplace signage and worked closely with each center on policy monitoring and enforcement planning. Finally, TTTF provided centers with health promotion materials that were designed to their specifications to reflect the demographics of the patients that they treated. These materials were provided in multiple languages and ranged from posters for offices to brochures for patients; materials advised patients to ask their healthcare provider for assistance with quitting tobacco and always included the Texas Tobacco Quitline phone number. Participating centers did not receive direct compensation for participation. All study procedures were approved by the Institutional Review Board at the University of Houston.

### 2.3. Survey Procedures

Data reported herein were collected via an online survey emailed to the providers within each center by their program champion, once before TTTF was implemented (pre-implementation) and once after all components of TTTF were implemented (post-implementation). Each survey request was open for ~3 weeks, and weekly reminders were sent for completion. Informed consent for participation was gathered via a survey cover letter; a waiver of documentation of informed consent was obtained. Data were collected anonymously to maintain provider privacy and encourage honest responses. As a result, pre- and post-implementation data could not be matched at the provider level. However, data could be matched at the center level. Due to the frequency of employee turnover at SUTCs [64,65,66,67], an unknown proportion of providers likely participated in the pre- but not the post-implementation procedures and vice versa.

### 2.4. Survey Measures

The survey assessed the primary variables of interest in the current study: providers’ perceived barriers to care, provider-reported education receipt, and provider-reported tobacco use disorder intervention practices. Each of these was assessed at pre-implementation and post-implementation.

Perceived barriers. Providers were asked: “What barriers do you face in regularly treating tobacco-using patients?” Of interest to this analysis were responses to the following barriers, each coded as yes or no: (1) lack of knowledge on how to treat tobacco with counseling and (2) lack of knowledge on how to treat tobacco with medications.

Education receipt. Education receipt was assessed with an item reading: “In the last 12 months, have you received any training on…” with two areas of interest, each coded as yes or no: (1) the use of counseling and behavior therapies to treat tobacco use and (2) the use of pharmacotherapies (e.g., NRT, Chantix) to treat tobacco use.

Intervention practices. Intervention practices were assessed with an item reading: “What types of treatment do you typically provide for cigarette smokers and/or other tobacco users?” Of interest to this analysis were endorsements of the following practices, each coded as yes or no: (1) behavioral counseling and (2) NRT (or referral/recommendation therefor) and/or non-nicotine medications (or referral therefor).

### 2.5. Statistical Analysis

First, center-level perceived provider knowledge barriers, education receipt, and intervention practice variables were derived from unmatched individual-level data for each center at pre- and post-implementation. Next, changes in each of these variables over time were investigated using chi-square/Fisher’s exact tests. Changes in center-level knowledge barrier variables were calculated as %/mean at post-implementation *minus* %/mean at pre-implementation. Median split binary center-level barrier variables were created for moderator analyses. Next, whether changes in education receipt and providers’ intervention practices over time differed by low versus high center-level changes in corresponding knowledge barriers to care provision was assessed with interaction terms. To account for the nested data structure of providers within the SUTCs, generalized linear mixed models (binomial distribution, logit link, and variance components for the variance matrix) were used.

All analyses were conducted using SAS Version 9.4 [68], and the level of significance was designated at *p* < 0.05.

## 3. Results

### 3.1. Provider-Reported Knowledge Barriers with Respect to Treating Patients with Behavioral Counseling and/or Medications

At pre-implementation, 20.46% of providers reported lack of knowledge on how to treat tobacco use with counseling as a barrier they face in regularly treating tobacco use; 26.64% of providers reported lack of knowledge on how to treat tobacco use with medications as a barrier they face regularly treating patients who use tobacco. At post-implementation, 9.28% of providers reported lack of knowledge on how to treat tobacco use with counseling as a barrier they face in regularly treating patients who use tobacco; 11.86% of providers reported lack of knowledge on how to treat tobacco use with medications as a barrier they face regularly treating patients who use tobacco. The decreases in these barriers over time with program implementation were statistically significant (*p*s < 0.05) in both cases and slightly more substantial for medication than counseling overall. See Table 1.

### 3.2. Provider-Reported Education Receipt and Intervention Practices

At pre-implementation, 32.00% of providers reported receipt of counseling and behavioral therapy education in the last year and 19.31% reported using behavioral counseling with patients who use tobacco; 20.46% of providers reported receipt of medication education in the last year and 31.66% reported using medications with patients who use tobacco or referring them for medications. At post-implementation, 70.21% of providers reported receiving counseling and behavioral therapy education in the last year and 28.87% reported using behavioral counseling with patients who use tobacco; 71.88% of providers reported receiving medication education in the last year and 55.15% reported using medications with patients who use tobacco or referring them for medication. These increases over time were significant in each case (*p*s < 0.05). Although education provided during TTTF program implementation reached a substantial proportion of providers (>70%) in both areas, changes in the use of medications or medication referral to address tobacco use with patients following education receipt were more substantial than those seen for counseling provision. See Table 2 and Table 3 for providers’ education receipt and intervention practices at pre- and post-program implementation by SUTC.

### 3.3. Changes in Lack of Knowledge of How to Treat Patients with Counseling as a Moderator of Changes in Provider-Reported Counseling Education Receipt and Associated Intervention Practices over Time

Contrary to hypotheses, there was no moderating effect of center-level changes in provider-reported knowledge barriers for counseling on changes over time in provider-reported receipt of counseling and behavioral therapy education (Estimate = 0.122, SE = 0.472, *p* = 0.797) or provider-reported regular use of behavioral counseling with patients (Estimate = −0.388, SE = 0.465, *p* = 0.405). (Table 4). Stated another way, high (versus low) changes in counseling knowledge being a barrier to care provision at the center level were not associated with increased receipt of education on cessation counseling or increased provision of cessation counseling to patients from pre- to post-program implementation.

### 3.4. Changes in Lack of Knowledge of How to Treat Patients with Medications or Refer Them for Medications as a Moderator of Changes in Provider-Reported Medication Education Receipt and Intervention Practices over Time

There was a moderating effect of center-level changes in provider-reported knowledge barriers of how to treat patients with or refer them for tobacco cessation medications on changes over time in provider-reported receipt of medication education (γ = 2.398, SE = 0.590, *p* < 0.001), and in provider-reported regular use of/referral for medications with patients who use tobacco (γ = 1.351, SE = 0.450, *p* = 0.003) (Table 4).

Specifically, further analyses showed that centers with high changes in the percentage of providers endorsing lack of knowledge about medications as a perceived barrier over time had increased odds of endorsing the receipt of medication education over time (OR from pre- to post-implementation: 0.170 to 10.345) compared to those from centers with low changes in the percentage of providers endorsing this perceived barrier over time (OR from pre- to post-implementation: 0.442 to 2.450). Stated another way, centers that experienced high reductions in the barrier of lack of knowledge of how to treat tobacco use with medications over time were more likely than those with low reductions (or increases) to report increases in the receipt of education in pharmacotherapies from pre- to post-program implementation; centers wherein providers had the least exposure to medication education prior to program implementation and took advantage of this education demonstrated commensurate decreases in reported knowledge barriers to medication usage.

Analyses additionally showed that centers with high changes in the percentage of providers endorsing lack of knowledge about medications as a perceived barrier to their use with patients from program implementation also had increased odds of using medications with patients or referring them for medication use over time (OR from pre- to post-implementation: 0.285 to 1.929) relative to centers with low changes in endorsement of this perceived barrier (OR from pre- to post-implementation: 0.750 to 1.313). Stated another way, centers that experienced high reductions in the barrier of lack of knowledge of how to treat or refer patients who use tobacco with medications over time also were more likely than those with low reductions (or increases) to report increases in the use of or referral for medications with patients who used tobacco from pre- to post-program implementation; centers wherein providers had the least exposure to medication education prior to program implementation and took advantage of this education demonstrated commensurate increases in medication cessation care provision with patients.

## 4. Discussion

The current study aimed to examine how education provision affected changes in providers’ self-reported knowledge barriers and their provision of tobacco cessation counseling and medication within 15 SUTCs in Texas, U.S., that served a nine-county catchment area through ~300,000 care visits annually. Ultimately, results were intended to inform the design of future SUTC workplace educational programs to maximize the provision of evidence-based tobacco use care to patients with SUDs, who are known to use tobacco at greater rates than the general population [11,12], experience commensurate disparities in tobacco-related morbidity and mortality [11,13], and may experience a lower likelihood of tobacco care receipt as a result of SUTC providers’ knowledge deficits [17,23,24,25,26]. Overall, results supported several hypotheses and can lend insight to SUTC workplace intervention planning for capacity building in tobacco use care provision to patients with SUDs.

Results reflected that knowledge gaps as barriers to care provision were generally low in participating SUTCs, endorsed overall by 20.46% (counseling) and 26.64% (medication) of providers within these settings. This may be somewhat surprising given various studies and theories citing knowledge dearth and misinformation as major factors underlying low rates of tobacco cessation care provision in SUTC settings [17,20,21,22,23,24,25,26]. Moreover, knowledge gaps hindering medication intervention/referral provision prior to program implementation were more common in participating SUTCs than knowledge gaps hindering cessation counseling provision. This was expected given that most providers in these settings are not medically trained and are thus less likely to know about medications for addressing tobacco use [46,47]. However, findings highlight the importance of covering medication usage and referral in educational interventions for providers in this setting, particularly as prescription privileges are unnecessary to provide patients with guidance on topics such as over-the-counter NRT use. The degree to which medication coverage is included and assessed in prior studies is often not reported [36,37,39]. The literature would benefit from future studies very clearly reporting the extent to which medication (and counseling) is covered and assessed in educational interventions. While education provision significantly reduced providers’ self-reported knowledge barriers for both cessation medication and counseling care provision from pre- to post-program implementation, it is noteworthy that such reduction was slightly more substantial for medication (−14.78%) than for counseling (−11.18%). This may not be surprising given, again, that most SUTC providers are not medically trained and were thus less likely to know about medications for addressing tobacco use at pre-implementation than they were to know about counseling [46,47]. This finding further highlights the importance of covering medication usage and referral in educational interventions.

Pre-implementation knowledge barriers were smaller than expected and, relatedly, so too were reductions in knowledge barriers following education provision, potentially calling into question the ability of educational interventions to translate into significant gains in cessation service delivery within SUTCs. Employee turnover from pre- to post-implementation may have contributed to the latter observation as well [64,65,66,67]. The counseling and medication knowledge barrier reductions, however, were accompanied by significantly enhanced delivery of counseling care at 13 of 15 SUTCs (19.31% to 28.87% overall) and cessation medication at 13 of 15 SUTCs (31.66% to 55.15% overall) from pre- to post-implementation. Thus, the present study not only provides support that provider-focused educational interventions can increase the provision of tobacco use care in various healthcare settings, similar to previously reported work [36,37,38], but also extends that notion to individual components of evidence-based tobacco use care—counseling and medication—in SUTCs. Results also support the importance of addressing providers’ knowledge gaps with education—even if they are only relevant to a fifth to a quarter of an SUTC’s providers. Implications include that programs designed to enhance the treatment of tobacco use disorder concurrently with the treatment of non-tobacco SUDs may therefore maximize their impact by including an educational component, or modifying their existing educational component, to spur knowledge absorption and/or implementing such programs at SUTCs with the greatest potential for medication knowledge gain (i.e., those with the most to learn or those who report the greatest barrier of lack of knowledge) [69]. Many strategies to improve knowledge absorption have been employed across various settings and contexts. Those strategies include making the education session interactive [70,71,72], leveraging prior knowledge [73], and using multiple methods/modalities for learning [74]. While there is potential for these strategies to improve knowledge absorption and consequently enhance tobacco treatment delivery to SUTC patients, more research is necessary to determine their effectiveness in this context and their relative effectiveness for training on counseling versus medication. Strategies to identify SUTCs with the greatest potential for knowledge gain may involve assessing institutional knowledge or educational resources prior to the implementation of educational interventions.

It is worth noting that greater enhancement in providers’ care provision was seen in cessation medication usage (+23.49%) than in counseling care provision (+9.56%). This result is also consistent with expectations and may be due to differences in the mechanisms underlying the uptake of the two treatment practices and/or in the logistics of delivering the two forms of treatment. As previously noted, many SUTC providers have advanced training in non-nicotine addiction counseling rather than medication usage [46,47], and tobacco cessation care information novelty has been linked to larger SUTC-level increases in tobacco use care provision and greater valuation of such provision by SUTC leadership [48]. Taken together, these results indicate that medication education may have spurred greater enhancement of treatment delivery than did counseling education because cessation medication information was more novel to the SUTC providers who participated in the study. Novelty of education about cessation interventions might be increased by introducing them in the context of lesser-known tobacco topics. For example, providers seemed, from anecdotal experience, very interested in learning more about e-cigarettes and other new tobacco products, creating a pathway for pairing this information directly with methods of evidence-based care provision that may apply to new as well as old tobacco products. Additionally, future educational intervention programs should consider highlighting the novel components of tobacco use counseling (relative to non-nicotine addiction counseling) in addition to the 5As (an evidence-based brief intervention model [75]) and other brief counseling techniques [76,77,78].

Additional reasons for the differential uptake of cessation medication usage versus counseling care may relate to providers’ perceived time restrictions [24,25], the skill required to provide effective counseling [42,43,44], and/or providers’ comfort with counseling provision [24,45]. Each of these factors may stifle counseling care provision but not cessation medication usage. SUTC providers have cited a lack of time to intervene in tobacco use as one of the reasons they do not commonly provide tobacco cessation care to their patients [24,25]. In supplying NRT and training providers on when and how to provide it to patients, the TTTF program empowered providers with all the tools necessary to efficiently provide medication to their patients. The TTTF program provided counseling training but did not make the provision of counseling more efficient. The fact that there were still overall increases in counseling provision suggests that, armed with increased knowledge of the benefits of counseling, some providers either made the time in their schedules for counseling or found ways to streamline individual patient visits to accommodate time for counseling. Doing either is difficult though, so time with patients may have a rate-limiting effect on counseling care uptake (i.e., at a certain point, additional counseling knowledge may not translate to additional counseling provision due to limited time to implement the acquired knowledge). Future educational interventions should consider placing increased emphasis on the efficiency and efficacy of brief counseling [76,77,78]. If providers are more aware that they can effectively counsel tobacco cessation with minimal time commitment, they may be more inclined to do so. At a minimum, providers should be educated on their local Quitline (i.e., eligibility for care, services provided therein, etc.).

Time constraints aside, adept medication delivery requires little more than the appropriate knowledge and access to over-the-counter NRT (and, in the case of bupropion, varenicline, other non-nicotine medications, and prescription NRTs (e.g., inhalers), a license to prescribe pharmaceutical agents). Adept counseling, on the other hand, is a skill that has been shown to improve with observation of model counseling, practice, and feedback [42,43,44]. Evidence suggests that, even with improved knowledge, providers may not increase their delivery of counseling without improved self-efficacy in doing so [69]. While the TTTF program invited SUTC providers to attend a ~7.5 h motivational interviewing training that included opportunities to observe, practice, and receive feedback, providers were not required to attend. The basic tobacco education that targeted all providers did not allow practice opportunities, as it was necessarily time-limited to facilitate availability and attendance, and topical coverage was prioritized over practice and coaching. Future educational interventions should partner with SUTC leadership to create opportunities for the observation and practice of tobacco cessation counseling care to build providers’ self-efficacy, even if only as follow-up programming to an educational session.

Still, even time and skill may not be sufficient to overcome inertia in providing counseling care. Studies have found that some providers do not discuss tobacco cessation with their patients because they are not comfortable having such potentially unpleasant conversations [24,45]. SUTC providers are likely to be more accustomed than most to having conversations about substance use cessation. Tobacco use cessation, however, may be a particularly uncomfortable topic to broach given that tobacco use is more socially accepted than other SUDs, especially in SUTC settings. As noted earlier, many SUTC providers even believe that tobacco use is a helpful facilitator of patients’ social connections [26]. While the latter can be addressed through education, improving knowledge on treating tobacco with counseling does not alone address the discomfort a provider may have in talking with patients about tobacco use cessation. Research has found that past unpleasant experiences underlie this discomfort [45], but future studies should explore methods to overcome this discomfort when treating SUTC patients who use tobacco. In the meantime, facilitating providers’ attempts to practice counseling (as discussed above in the context of improving skill) may alleviate some of their discomfort. Specific efforts to address the discomfort of tobacco-using providers arising from their personal use should also be considered [18,19].

Although most studies on the effect of education on tobacco cessation care delivery in healthcare settings examine factors on the individual provider level [36,37,38], when considering non-profit SUD community care, it is important to consider SUTC-level gains in cessation knowledge and care provision. These SUTCs may represent the affordable and accessible care options that cover an entire geographic catchment coverage area. Thus, center-level capacity development is of interest as it has the potential to affect area-level tobacco-related health inequities. The current study found that the magnitude of changes at the SUTC level in knowledge barriers over time consequent to education receipt moderated changes in medication provision/referral but not in counseling care provision. All of the aforementioned reasons for the differential uptake of cessation medication usage versus counseling care (i.e., information novelty, perceived time restrictions, skill, and comfort) may have contributed to this result. For example, the potential rate-limiting effect of time with patients on counseling provision may inhibit the magnitude of reduction in counseling knowledge barriers from translating into increased provision of counseling care. This further highlights the need for future educational interventions to emphasize the novelty and effectiveness of brief counseling techniques and to give providers opportunities to develop skill and comfort with those techniques through practice. Moreover, examination of capacity building at the SUTC level is also recommended, particularly when targeting SUTCs serving rural or medically underserved patients who may live in places with elevated tobacco use rates and lower access to evidence-based cessation care [50].

This study was intentionally limited to the education of SUTC providers, with the goal of uncovering ways to enhance the provision of tobacco use treatment to SUTC patients who bear a disproportionate burden of tobacco-related disease and death. Future studies may wish to investigate educating other professionals (e.g., pharmacists) who commonly interact with individuals who use tobacco products. Other limitations of the present study include that not all providers at the participating SUTCs were reached by the surveys or the educational intervention, that some gaps in knowledge were apparently unaddressed, and that a notable portion of participating providers may currently use tobacco or have used it in the past [18,19]. Providers were not required to complete the pre-implementation survey, participate in TTTF, or complete the post-implementation survey. Results are extrapolated to entire SUTCs based on the providers that opted to participate, which may introduce selection bias. Future educational intervention programs may incentivize participation by providing relevant continuing education credits and/or encouraging SUTC leadership to factor participation into provider compensation (e.g., merit raises). While TTTF implementation reduced knowledge barriers, some counseling and medication knowledge barriers persisted post-implementation (9.28% and 11.86%, respectively). This may be due to employee turnover between surveys or to some providers taking the post-implementation survey without attending the educational sessions, but it likely at least partially reflects that some providers did not find that the TTTF educational sessions eliminated their knowledge gaps. Future iterations of TTTF should obtain feedback from educational session participants to better understand what knowledge gaps persist and how to address them. This feedback obtainment may include inviting participants to give critical input via qualitative procedures. The TTTF educational program included broadly applicable information but was not tailored to reach SUTC providers who themselves use tobacco. Future work should investigate how providers’ tobacco use status may affect their participation in non-compulsory tobacco cessation education sessions and how educational intervention programs may overcome such reluctance to treat tobacco use. Finally, given increased recognition of the complexity of environments, future studies may wish to leverage machine learning techniques in their data collection and analyses [79].

## 5. Conclusions

This study found that, following an educational intervention to increase knowledge on treating tobacco use with counseling and/or medication, SUTC providers demonstrated increased provision of those respective treatments to their patients who use tobacco. Centers with the greatest reductions in the barrier of lack of knowledge on treating tobacco use with medication were more likely to report greater increases in the provision of medication (or referral therefor) to their patients who use tobacco. The magnitude of center-level knowledge change did not moderate the magnitude of increases in the provision of counseling or behavioral therapy. This work adds to the existing literature by (1) demonstrating that educational intervention can enhance the specific provision of counseling and medication to treat tobacco use among SUTC patients and (2) evidencing the differential moderating effect of center-level knowledge gain on treatment provision. This information can help to tailor time-limited educational interventions to an SUTC’s needs and maximize its potential to impact tobacco use disparities in a given community. Uptake of counseling education may be better actualized if brief counseling is emphasized and/or if educational sessions require that providers attend and practice counseling skills. These findings also highlight areas for future study, including how to overcome provider discomfort with tobacco cessation conversations.

## Figures and Tables

**Table 1:** Changes in providers’ perceived lack of knowledge barriers for tobacco cessation care provision from pre-to post-implementation of a comprehensive tobacco-free workplace program that included education in 15 substance use treatment centers in Texas (pre-implementation *n* = 259 providers; post-implementation *n* = 194 providers).

	Barrier of Lack of Knowledge on Using Behavioral Counseling					Barrier of Lack of Knowledge on Using Medication				
	Pre %	Pre N	Post %	Post N	*p*-Value	Pre %	Pre N	Post %	Post N	*p*-Value
SUTC 1	20.00	25	4.55	22	0.1936	20.00	25	4.55	22	0.1936
SUTC 2	0.00	3	0.00	2	NA	0.00	3	50.00	2	0.4000
SUTC 3	0.00	6	0.00	4	NA	16.67	6	0.00	4	1.0000
SUTC 4	0.00	11	15.38	13	0.4819	36.36	11	0.00	13	0.0311
SUTC 5	33.33	3	0.00	4	0.4286	0.00	3	25.00	4	1.0000
SUTC 6	36.00	50	10.00	40	0.0043	40.00	50	2.50	40	<0.0001
SUTC 7	13.04	23	10.53	19	1.0000	26.09	23	26.32	19	1.0000
SUTC 8	66.67	3	0.00	2	0.4000	66.67	3	50.00	2	1.0000
SUTC 9	26.15	65	14.00	50	0.1119	23.08	65	18.00	50	0.5066
SUTC 10	37.50	8	0.00	1	1.0000	50.00	8	0.00	1	1.0000
SUTC 11	0.00	3	0.00	5	NA	33.33	3	20.00	5	1.0000
SUTC 12	3.33	30	7.14	14	0.5402	23.33	30	14.29	14	0.6951
SUTC 13	22.22	9	14.29	7	1.0000	0.00	9	14.29	7	0.4375
SUTC 14	0.00	11	0.00	3	NA	18.18	11	0.00	3	1.0000
SUTC 15	11.11	9	0.00	8	1.0000	22.22	9	0.00	8	0.4706
All	20.46	259	9.28	194	0.0012	26.64	259	11.86	194	0.0001

Note: SUTC = substance use treatment center.

**Table 2:** Changes in provider-reported past-year education receipt for counseling and behavioral therapy for tobacco and the regular use of counseling and behavioral therapy with patients who use tobacco from pre- to post-implementation of a comprehensive tobacco-free workplace program that provided education on cessation counseling in 15 substance use treatment centers in Texas (pre-implementation *n* = 259 providers; post-implementation *n* = 194 providers).

	Receipt of Counseling and Behavioral Therapy Education					Behavioral Counseling Used with Patients				
	Pre %	Pre N	Post %	Post N	*p*-Value	Pre %	Pre N	Post %	Post N	*p*-Value
SUTC 1	30.43	23	45.45	22	0.2989	20.00	25	31.82	22	0.3538
SUTC 2	66.67	3	100.00	2	1.0000	66.67	3	100.00	2	1.0000
SUTC 3	33.33	6	100.00	4	0.0762	16.67	6	50.00	4	0.5000
SUTC 4	45.45	11	92.31	13	0.0233	36.36	11	38.46	13	1.0000
SUTC 5	33.33	3	75.00	4	0.4857	0.00	3	25.00	4	1.0000
SUTC 6	12.00	50	78.38	37	<0.0001	6.00	50	12.50	40	0.4584
SUTC 7	52.17	23	64.71	17	0.4280	34.78	23	47.37	19	0.4082
SUTC 8	33.33	3	0.00	1	1.0000	0.00	3	50.00	2	0.4000
SUTC 9	27.87	61	58.00	50	0.0013	10.77	65	24.00	50	0.0582
SUTC 10	37.50	8	100.00	1	0.4444	25.00	8	100.00	1	0.3333
SUTC 11	50.00	2	80.00	5	1.0000	33.33	3	20.00	5	1.0000
SUTC 12	40.00	30	85.71	14	0.0046	33.33	30	35.71	14	1.0000
SUTC 13	50.00	8	100.00	7	0.0769	11.11	9	14.29	7	1.0000
SUTC 14	36.36	11	66.67	3	0.5385	18.18	11	33.33	3	1.0000
SUTC 15	37.50	8	75.00	8	0.3147	44.44	9	37.50	8	1.0000
All	32.00	250	70.21	188	<0.0001	19.31	259	28.87	194	0.0174

Note: SUTC = substance use treatment center.

**Table 3:** Changes in provider-reported past-year education receipt on the use of or referral for pharmacotherapies for tobacco use and the regular use of recommendation/referral for medication with patients who use tobacco from pre- to post-implementation of a comprehensive tobacco-free workplace program that provided education on medication use/referral in 15 substance use treatment centers in Texas (pre-implementation *n* = 259 providers; post-implementation *n* = 194 providers).

	Receipt of Medication Education					Medication Use/Referral with Patients ^§^				
	Pre %	Pre N	Post %	Post N	*p*-Value	Pre %	Pre N	Post %	Post N	*p*-Value
SUTC 1	20.00	25	54.55	22	0.0139	36.00	25	72.73	22	0.0118
SUTC 2	66.67	3	100.00	2	1.0000	66.67	3	100.00	2	1.0000
SUTC 3	0.00	6	100.00	4	0.0048	16.67	6	100.00	4	0.0476
SUTC 4	36.36	11	100.00	13	0.0010	27.27	11	69.23	13	0.0405
SUTC 5	33.33	3	50.00	4	1.0000	33.33	3	75.00	4	0.4857
SUTC 6	4.00	50	78.95	38	<0.0001	12.00	50	37.50	40	0.0045
SUTC 7	13.04	23	63.16	19	0.0007	34.78	23	31.58	19	0.8265
SUTC 8	33.33	3	50.00	2	1.0000	0.00	3	50.00	2	0.4000
SUTC 9	27.69	65	62.00	50	0.0002	46.15	65	52.00	50	0.5341
SUTC 10	37.50	8	100.00	1	0.4444	12.50	8	100.00	1	0.2222
SUTC 11	33.33	3	80.00	5	0.4643	66.67	3	80.00	5	1.0000
SUTC 12	13.33	30	64.29	14	0.0011	33.33	30	21.43	14	0.4984
SUTC 13	77.78	9	100.00	7	0.4750	22.22	9	100.00	7	0.0032
SUTC 14	9.09	11	100.00	3	0.0110	36.36	11	100.00	3	0.1923
SUTC 15	11.11	9	87.50	8	0.0034	33.33	9	87.50	8	0.0498
All	20.46	259	71.88	192	<0.0001	31.66	259	55.15	194	<0.0001

Note. ^§^ Nicotine replacement therapy (e.g., nicotine patch, gum) or referral/recommendation for such and/or non-nicotine medications (e.g., Chantix) or referral for such. SUTC = substance use treatment center.

**Table 4:** Examination of center-level changes in lack of knowledge as a barrier to care provision as a moderator of changes in provider-reported receipt of education and provider-reported intervention practices from before to after the implementation of a comprehensive tobacco-free workplace program in 15 Texas substance use treatment centers.

Outcome	Effect	Estimate	SE	*p*-Value
Receipt of Counseling and Behavioral Therapy Education	Time (ref: pre-implementation)	1.683	0.264	<0.0001
	Barrier: Lack of Knowledge on Treating Tobacco Use with Counseling *	0.920	0.279	0.001
	Interaction Term	0.122	0.472	0.797
Behavioral Counseling Used with Patients	Time (ref: pre-implementation)	0.760	0.317	0.017
	Barrier: Lack of Knowledge on Treating Tobacco Use with Counseling *	0.878	0.432	0.043
	Interaction Term	−0.388	0.465	0.405
Receipt of Medication Education	Time (ref: pre-implementation)	1.714	0.270	<0.0001
	Barrier: Lack of Knowledge on Treating Tobacco Use with Medications ^§,^*	−0.957	0.606	0.1148
	Interaction Term	2.398	0.590	<0.0001
Medication Used or Referral for Medication Used with Patients	Time (ref: pre-implementation)	0.560	0.246	0.023
	Barrier: Lack of Knowledge on Treating Tobacco Use with Medications ^§,^*	−0.967	0.446	0.031
	Interaction Term	1.351	0.450	0.003

* The reference group for knowledge barriers was low (versus high) changes over time. ^§^ Nicotine replacement therapy (e.g., nicotine patch, gum) or referral/recommendation for such and non-nicotine-based medications (e.g., Chantix) or referral for such.

## Data Availability

The data presented in this study are available on request from the corresponding author. The data are not publicly available due to confidentiality/privacy agreements with the funders.
